# Supplementary figures and images for: Comparison of hybrid learning and remote education in the implementation of the “Adopt a Microorganism” methodology
Source: PLoS One. 2021 Nov 24;16(11):e0248906. doi: 10.1371/journal.pone.0248906 (PMC8612561; doi:10.1371/journal.pone.0248906)

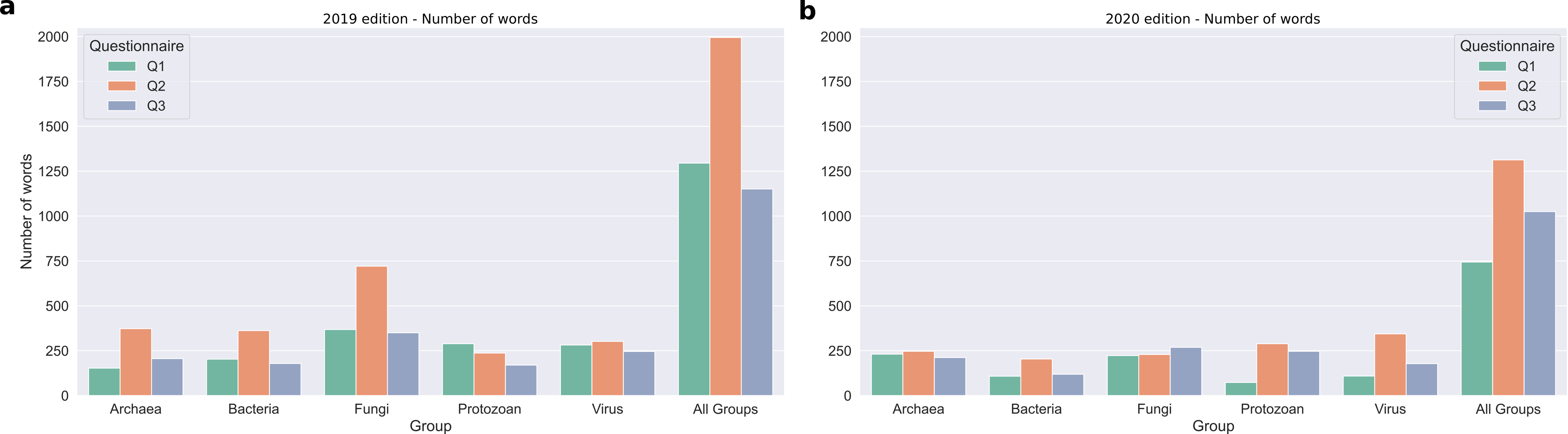

Supplement: S1 Fig — [a] 2019 edition; [b] 2020 edition. (TIF) [file pone.0248906.s001.tif]

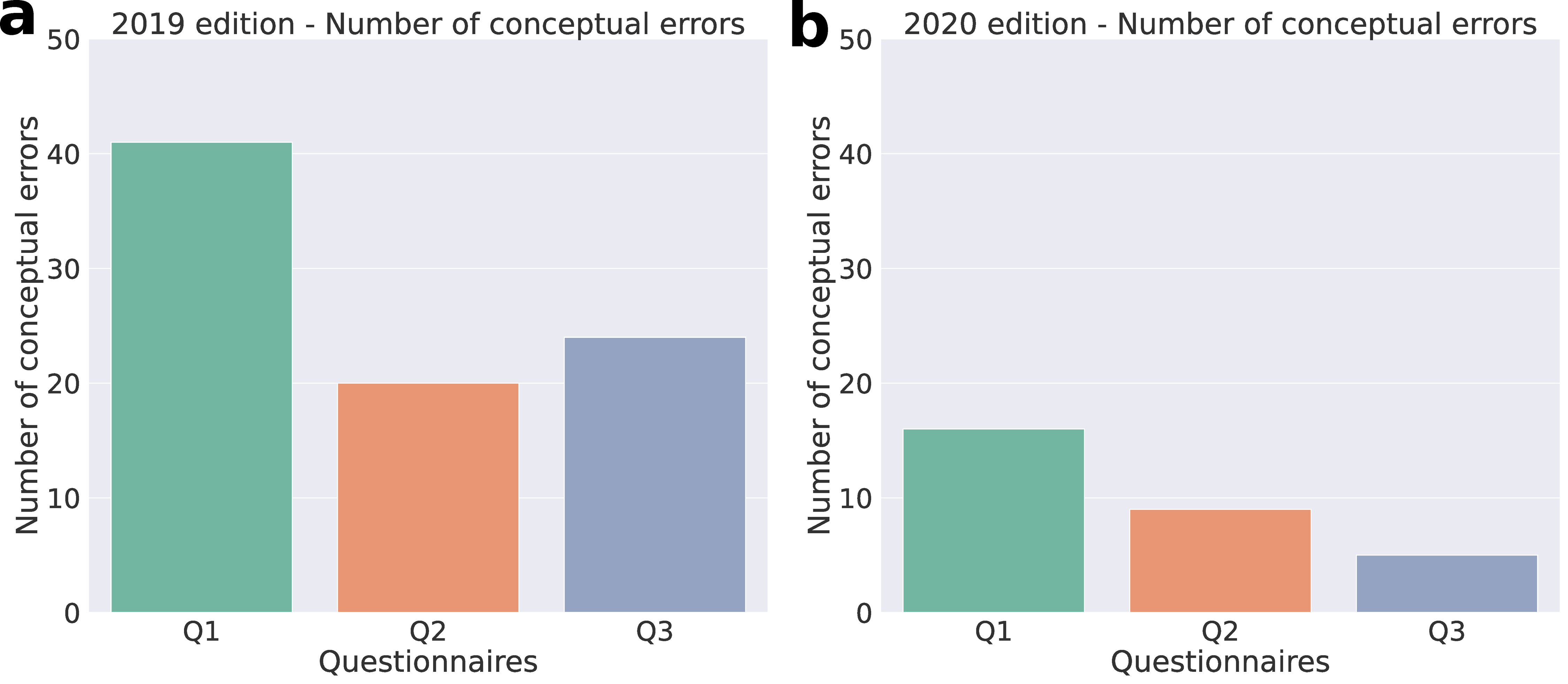

Supplement: S2 Fig — [a] 2019 edition; [b] 2020 edition. (TIF) [file pone.0248906.s002.tif]

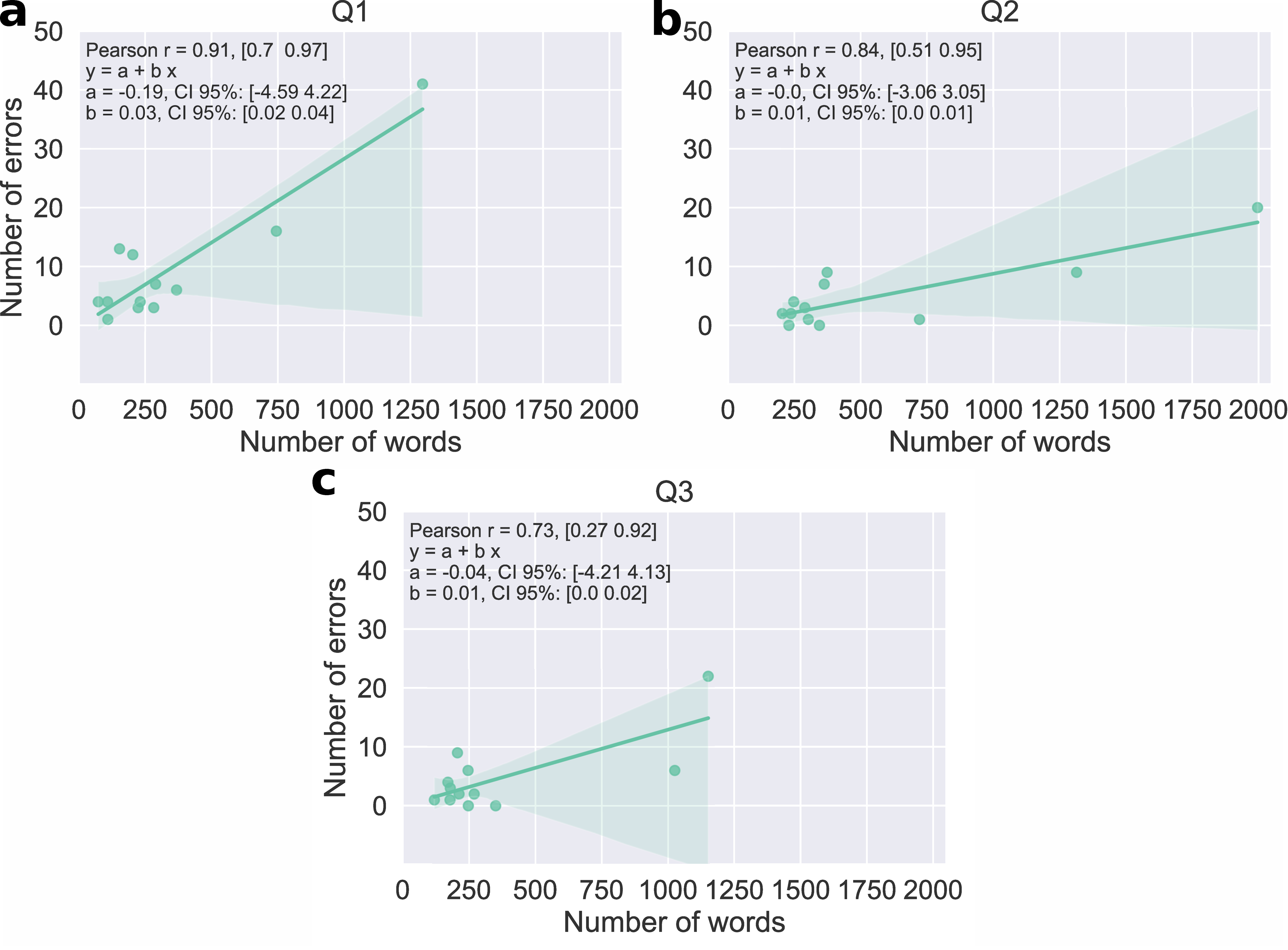

Supplement: S3 Fig — In each plot is indicated the Pearson’s correlation coefficient and its confidence interval; as well as the linear regression model, with the intercept (a) and slope (b) terms and their respective confidence interval. [a] Q1 questionnaire; [b] Q2 questionnaire and [c] Q3 questionnaire. (TIF) [file pone.0248906.s003.tif]

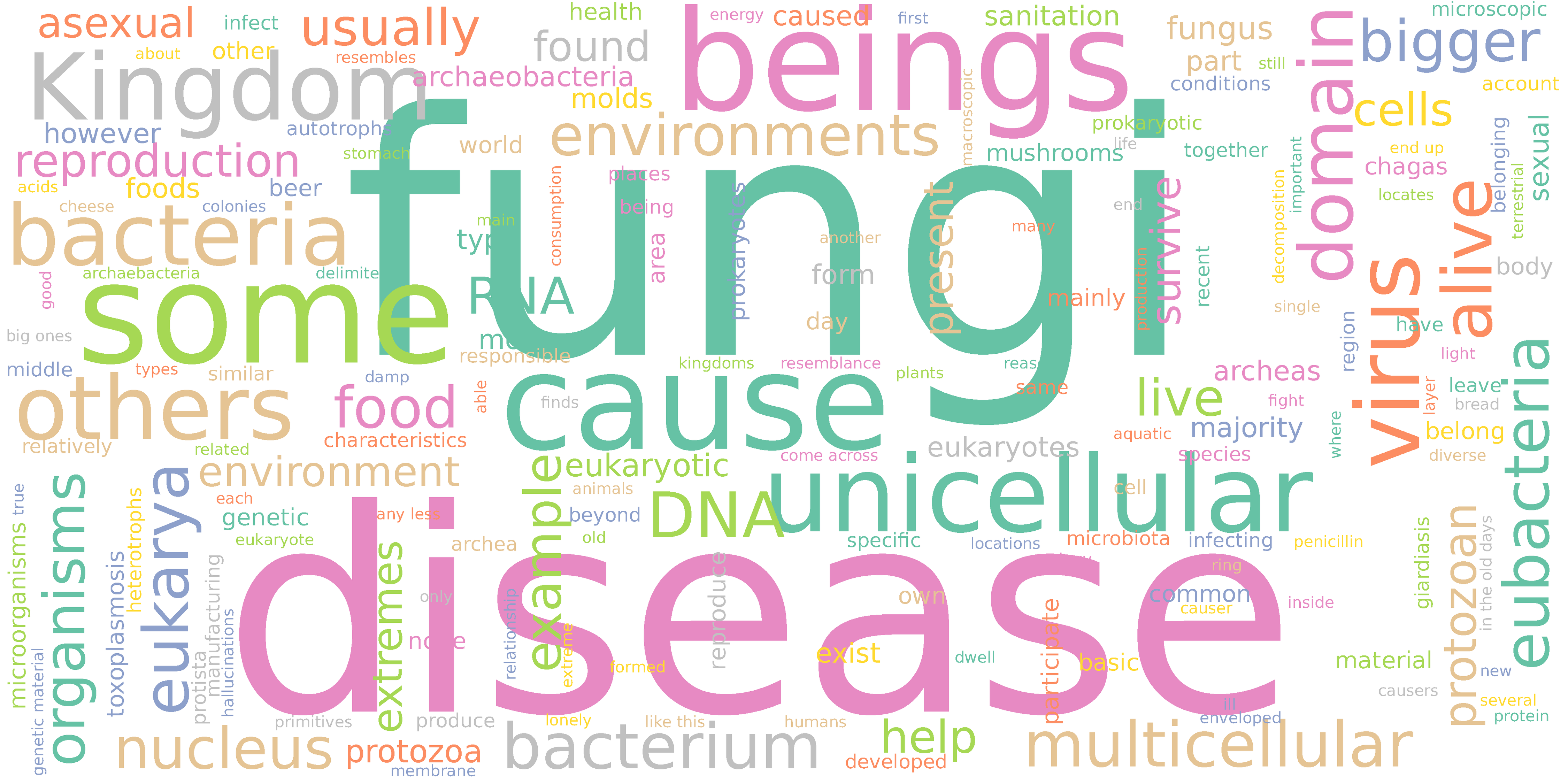

Supplement: S4 Fig — (TIF) [file pone.0248906.s004.tif]
