# Supplementary material for: Comparison of hybrid learning and remote education in the implementation of the “Adopt a Microorganism” methodology
Source: PLoS One. 2021 Nov 24;16(11):e0248906. doi: 10.1371/journal.pone.0248906 (PMC8612561; doi:10.1371/journal.pone.0248906)
Supplement: S2 Appendix — (PDF) [file pone.0248906.s009.pdf]

ADOPT A MICROORGANISM PROJECT EVALUATION QUESTIONNAIRE

1- Please indicate, according to your opinion, the positive points in using Face-book® as a tool to learn about microorganisms.

2- Now indicate the negative points in the use of Facebook®.

3- How do you evaluate the participation of the mediators?

4- How could mediators improve their participation?

5- Below are some statements (A to D) related to the use of Facebook® in the learning process. Evaluate how much each of these statements contributed to your learning about Biology in the Adopt a Microorganism project. For this evaluation, use the following scale:

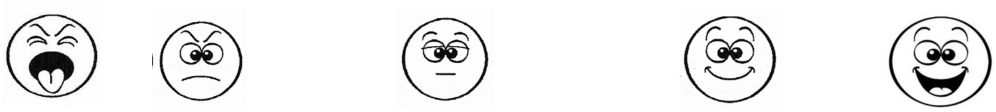

Terrible Bad	Bad	More or Less	Good	Excellent

A- I was able to learn and study when and where I wanted.

B- I had more contact with classmates, teachers and the mediators to clarify doubts about the subject.

C- I was able to participate more actively in my own learning.

D- Easier to ask questions than in the classroom.

6- We're done! Would you like to comment on anything else? Be my guest!
